# Characterization of feedback-resistant mevalonate kinases from the methanogenic archaeons *Methanosaeta concilii* and *Methanocella paludicola*

**DOI:** 10.1099/mic.0.000510

**Published:** 2017-09-05

**Authors:** Ekaterina Kazieva, Yoko Yamamoto, Yoshinori Tajima, Keiichi Yokoyama, Joanna Katashkina, Yousuke Nishio

**Affiliations:** ^1^​ Ajinomoto-Genetika Research Institute, Moscow, Russia; ^2^​ Institute for Innovation, Ajinomoto Co., Inc., Kanagawa, Japan; ^3^​ R&D Planning Department, Ajinomoto Co., Inc., Tokyo, Japan

**Keywords:** mevalonate kinase, GHMP superfamily, feedback-resistant, mevalonate pathway

## Abstract

The inhibition of mevalonate kinase (MVK) by downstream metabolites is an important mechanism in the regulation of isoprenoid production in a broad range of organisms. The first feedback-resistant MVK was previously discovered in the methanogenic archaeon *Methanosarcinamazei*. Here, we report the cloning, expression, purification, kinetic characterization and inhibition analysis of MVKs from two other methanogens, *Methanosaetaconcilii* and *Methanocellapaludicola*. Similar to the *M. mazei* MVK, these enzymes were not inhibited by diphosphomevalonate (DPM), dimethylallyl diphosphate (DMAPP), isopentenyldiphosphate (IPP), geranylpyrophosphate (GPP) or farnesylpyrophosphate (FPP). However, they exhibited significantly higher affinity to mevalonate and higher catalytic efficiency than the previously characterized enzyme.

## Introduction

Isoprenoids, or terpenoids, are a very large class of naturally occurring compounds with diverse structures and functions. All are synthesized from the same five-carbon common precursors, isopentenyl diphosphate (IPP) and dimethylallyl diphosphate (DMAPP). Many isoprenoid compounds exhibit physiological activity. The spectrum of naturally produced isoprenoids, which includes carotenoids, steroid hormones, phytols, redox carriers, secondary metabolites and pheromones, is species-specific.

Two pathways for IPP and DMAPP biosynthesis have been discovered: the mevalonate (MVA) pathway and the 2-C-methyl-d-erythritol 4-phosphate (MEP) pathway ([1–3]; for a review see [4]).^13^ C NMR studies have shown that both acetate and mevalonate are precursors for isoprenoids in *Archaea* [5, 6]. These results were consistent with the subsequent analysis of archaeal genomes, which revealed homologues of mevalonate pathway enzymes, including 3-hydroxy-3-methylglutaryl-coenzyme A (HMG-CoA) synthase, HMG-CoA reductase [7–9], mevalonate kinase (MVK) and IPP isomerase [10, 11], but did not find homologous MEP pathway enzymes. Since putative genes encoding phosphomevalonate kinase (PMK) and diphosphomevalonate decarboxylase (DMD) were not identified on the basis of sequence similarity in archaeal genomes, while isopentenyl phosphate kinase (IPK) genes have been found in nearly all archaea, it has been hypothesized that an alternative mevalonate pathway operates in *Archaea* [12]. In this hypothetical pathway, phosphomevalonate is decarboxylated to yield isopentenyl phosphate (IP), which is then phosphorylated to IPP by the action of IPK. Recently, the missing phosphomevalonate decarboxylase (PMD) enzyme that converts (R)-mevalonate 5-phosphate (MVA-5-P) into IP was discovered in exceptional phyla of bacteria (*Chloroflexi*) and archaea (*Halobacteria*) [13–15]. Furthermore, MVA-3-P, but not MVA-5-P, serves as a precursor for IP synthesis in *Thermoplasma acidophilum*; a novel enzyme, ATP:(R)-MVA 3-phosphotransferase Ta1305, was identified in this species [16].

Organisms use a variety of regulatory mechanisms to ensure the sufficient formation of necessary isoprenoids and prevent the over-production of potentially toxic compounds (e.g. cholesterol). The inhibition of MVK by downstream metabolites such as DMAPP, IPP, GPP and FPP (class I MVKs) [17–20], or by diphosphomevalonate (class II MVKs) [21], plays an important role in the regulation of the mevalonate pathway in different organisms. The strict control of this reaction prevents the accumulation of phosphomevalonate, which can block DNA replication and arrest the cell cycle [22]. The first thermostable archaeal MVK to be biochemically characterized was from *Methanocaldococcus jannaschii*; inhibition by GPP and FPP, but not IPP, was observed [11].

Exploring the efficient feedback-resistant enzymes of the mevalonate pathway and their introduction into microbial-producing strains is a very important step towards the industrial production of isoprenoids. The discovery of a feedback-resistant MVK in the methanogenic archaeon *Methanosarcina mazei* [23] not only distinguished a new class of MVKs, but significantly broadened the opportunities for microbial isoprenoid production. Unlike most bacteria and eukaryotes, archaea equip their own membrane phospholipid structure, which is composed of isoprenoid chains condensed with sn-glycerol-1-phosphate by ether linkages (for reviews, see [24–26]). High-performance constitutive biosynthesis of the common precursors of isoprenoids can be expected in *Archaea* because they are building blocks of abundant components of archaeal membranes, which is why we would expect that the feedback-resistant MVK from *M. mazei* is not unique. The feedback resistance of the mevalonate pathway enzymes can thus be proposed as a common feature of large taxa belonging to *Archaea*.

We report here the cloning and overexpression of the *mvk* genes from *Methanosaeta concilii*, an archaeon belonging to the same order, *Methanosarcinales,* as *M. mazei*, from an evolutionarily distant methanogenic archaeon, *Methanocella paludicola*, and from *Nitrosopumilus maritimus*, an unrelated species belonging to *Thaumarchaeota*, in *Escherichia coli*; the purification, kinetic characterization and inhibition analysis of the corresponding enzymes are also described. The feedback-regulated *Saccharomyces cerevisiae* MVK and feedback-resistant *M. mazei* MVK were recharacterized in this study and used as controls. Our results suggest that *M. concilii*, *M. paludicola* and *N. maritimus* MVKs are feedback-resistant; the catalytic efficiencies of the first and second enzymes are higher than that of the MVK from *M. mazei*. Hereafter, the MVKs from *M. mazei*, *M. concilii*, *M. paludicola*, *N. maritimus* and *S. cerevisiae* will be referred to as MVK*^mma^*, MVK*^mcl^*, MVK*^mpd^*, MVK*^nmr^* and MVK*^sce^*, respectively.

## Methods

### Chemicals and reagents

Isopropyl thiogalactoside (IPTG), phosphoenolpyruvate (PEP), MgCl_2_, NaCl, lactate dehydrogenase, pyruvate kinase, (R)-lithium mevalonate, GPP ammonium salt, FPP ammonium salt, (±)-mevalonic acid 5-diphosphate tetralithium salt (DPM), HEPES and dithiothreitol (DTT) were purchased from Sigma-Aldrich (USA). DMAPP was obtained from Cayman Chemicals. Tris-HCl (pH 7.6) was purchased from WAKO (Japan). NADH and ATP were obtained from Oriental Yeast Co., Ltd. Mevalonate was prepared from mevalonolactone (ADEKA, Japan) as follows: 260 mg of mevalonolactone was mixed well with 5 ml of water and then with 0.6 ml of 10 N KOH. The mixed solution was then incubated at 37 °C for 2 h. After incubation, the pH of the solution was adjusted to 8.0 by neutralization with hydrochloric acid. The solution was then brought up to a final volume of 20 ml to yield a 100 mM potassium mevalonate solution. The obtained mevalonate solution was verified by HPLC. HPLC analysis was performed using 50 mM (R)-lithium mevalonate as the standard. The HPLC used for verification was a Hitachi high-performance liquid chromatograph (L-2000). HPLC was performed using YMC-Pack ODS-A (150×4.6 mm I.D. YMC, Japan) as the stationary phase and phosphoric acid (pH 2.5) as the mobile phase. Detection was performed at 210 nm.

### Bacterial strains and plasmid vectors

The pET-21a(+) (for the C-terminal His-tag) and pET- 28b(+) (for the N-terminal His-tag) vectors were used to express the recombinant protein. The recombinant plasmids were transformed into *E. coli* BL21 (DE3) cells for expression. The *E. coli* BL21 (DE3) strain and expression vectors were purchased from Novagen.

### Construction of expression plasmids

DNA fragments containing coding parts of the *mvk^mma^*, *mvk^mpd^*, *mvk^mcl^* and *mvk^nmr^* genes linked to ribosome-binding sites were chemically synthesized and amplified by PCR using the primers listed in Table 1. The forward and reverse primers contained *NdeI* and *HindIII* (*EcoRI* for *mvk*^mpd^) recognition sites (underlined), respectively.

**Table 1.** Primers used for cloning the *mvk* genes

Plasmid	Gene	Name	Sequence
pET-28b(+)	*mvk^mcl^*	P1	GCAATTCCATATGACGATGGCTTCCGCTCCGGGCAA
		P2	CCCAAGCTTACGCGACTTCCAGGCGAACACCTT
	*mvk^mpd^*	P3	GCAATTGCATATGACGATGTGCTCAGCTCCGGGTAA
		P4	GGAATTCACTGAATGAAAATACCTTCCGCCGTCGG
	*mvk^mma^*	P5	GCAATTCCATATGGTATCCTGTTCTGCGCCGG
		P6	CCCAAGCTTAATCTACTTTCAGACCTTGCTCGGTC
	*mvk^sce^*	P7	GCAATTGCATATGTCATTACCGTTCTTAACTTCTGC
		P8	CCCAAGCTTATGAAGTCCATGGTAAATTCGTGTT
	*mvk^nmr^*	P9	GCAATTCCATATGAAGAGCAAGGCATCTGCGCC
		P10	CCCAAGCTTAAAACGTGTCCAGGCCCTTGAAATC
pET-21a(+)	*mvk^mcl^*	P1	GCAATTCCATATGACGATGGCTTCCGCTCCGGGCAA
		P11	CCCAAGCTTCGCGACTTCCAGGCGAACACCTT
	*mvk^mpd^*	P3	GCAATTCCATATGACGATGTGTTCAGCCCCCGGTAA
		P12	CCCAAGCTTTTGGATGAATATTCCCTCCGCCGTT
	*mvk^mma^*	P5	GCAATTCCATATGGTATCCTGTTCTGCGCCGG
		P13	CCCAAGCTTATCTACTTTCAGACCTTGCTCGGTC
	*mvk^sce^*	P7	GCAATTGCATATGTCATTACCGTTCTTAACTTCTGC
		P14	CCCAAGCTTATGAAGTCCATGGTAAATTCGTGTT
	*mvk^nmr^*	P9	GCAATTCCATATGAAGAGCAAGGCATCTGCGCC
		P15	CCCAAGCTTAAACGTGTCCAGGCCCTTGAAATC

The ERG12 mevalonate kinase gene was amplified by PCR with Prime Star polymerase (Takara Bio Inc., Japan) using genomic DNA isolated from *S. cerevisiae* as a template.

The obtained DNA fragments were digested with *Nde*I and *Hind*III (*EcoRI* for *mvk^mpd^*) and inserted into the corresponding sites of pET-21a(+) and pET-28b(+). The expected DNA sequences in the inserted DNA fragments were confirmed by sequencing.

### Expression and preliminary mevalonate kinase activity assay

Each transformant was cultured at 20 °C by reciprocal shaking at 140 r.p.m. in Luria–Bertaini (LB) broth [27] containing 0.1 mg ml^−1^ ampicillin [for pET-21a(+)] or 0.05 mg ml^−1^ kanamycin [for pET-28b(+)]. When the OD_600_ reached approximately 0.5, IPTG was added to a final concentration of 1 mM, and cultivation was continued overnight at 20 °C. Cells were harvested by centrifugation and resuspended in buffer solution containing 50 mM sodium phosphate, 0.3 M NaCl and 20 mM imidazole. The cells were then disrupted by an ultrasonic disruptor (TOMY UD-201) at an output level of 3.5. After centrifugation (20 000 ***g***, 20 min), disrupted cells were adsorbed onto a His-SpinTrap column (GE Healthcare). After washing with the same buffer solution, the proteins were eluted with buffer solution containing 50 mM sodium phosphate, 0.3 M NaCl and 0.5 M imidazole (pH 7.5). The eluate was dialyzed overnight at 4 °C, with the external solution containing 20 mM Tris-HCl (pH 7.5) and 50 mM NaCl. The enzyme activity of each eluate in the internal dialysis solution was measured.

### Expression and purification of recombinant *M. concilii*, *M. paludicola*, *M. mazei, N. maritimus* and *S. cerevisiae* MVKs

To analyse the expression of MVKs derived from *M. mazei, N. maritimus* and *S. cerevisiae*, each transformant was cultured at 20 °C in a thick test tube containing 20 ml of LB broth by reciprocal shaking at 140 r.p.m. To analyse the expression of MVKs derived from *M. concilii* and *M. paludicola*, each transformant was cultured at 20 °C in 2 L LB broth in a 3 L shaking flask with baffles by gyratory culture at 140 r.p.m. When the OD_600_ reached approximately 0.5, IPTG was added to a final concentration 0.1 mM (for *mvk^mma^*, *mvk^nmr^* and *mvk^sce^*) or 1 mM IPTG (for *mvk^mpd^* and *mvk^mcl^*), and the cultures were incubated at 20 °C overnight to induce the target protein. Harvested cells were suspended in buffer solution A (50 mM sodium phosphate, 0.3 M NaCl and 20 mM imidazole), and the cells were disrupted with an ultrasonic disruptor. Ultrasonic disruption of the transformants expressing MVK*^mma^*, MVK*^nmr^* and MVK*^sce^* was performed at an output level of 3.5, while for the transformants expressing MVK*^mcl^* and MVK*^mpd^* it was performed at an output level of 8. After centrifugation (28 000 ***g***, 30 min), the supernatant was adsorbed onto a His-Trap HP (GE Healthcare) column to elute the target protein by a linear concentration gradient of imidazole (final concentration 0.5 M). The obtained proteins were dialyzed against an external solution containing 20 mM Tris-HCl (pH 8.0), 1 mM DTT and 50 mM NaCl. The dialyzed protein was designated as the purified enzyme. Protein quantity was measured with a Bio-Rad protein assay kit and a BSA standard.

### Preparation of PMK

The ERG8 gene (NM_001182727.1) encoding the phosphomevalonate kinase in *Saccharomyces cerevisiae* was amplified by PCR containing PrimeSTAR MAX DNA polymerase premix (Takara Bio, Inc.) using the primers 5′-TCAGAGTTGAGAGCCTTCAGTGCCCCAG-3′ and 5′-GGAATTCTCTTTATCAAGATAAGTTTCCGGATCTTTTT-3′ and genomic DNA from *S. cerevisiae* as a template. pET21-d(+) was digested with *NcoI*, treated with the Klenow fragment of DNA polymerase I, and then digested with *EcoRI*. The PCR-generated DNA fragment was digested with *EcoRI* and ligated with the described vector fragment. The expected DNA sequence of the inserted fragment was confirmed by sequencing.

*E. coli* BL21(DE3) was transformed with the obtained plasmid and cultured in 20 ml of LB broth at 30 °C by reciprocal shaking at 140 r.p.m. When the OD_600_ reached approximately 0.7, 0.1 mM IPTG was added and cultivation was continued overnight under the same conditions. Cells were harvested by centrifugation, resuspended in buffer solution A (50 mM sodium phosphate, 0.3 M NaCl and 20 mM imidazole) and disrupted by ultrasonication. After centrifugation, the resulting supernatant was adsorbed onto a His SpinTrap (GE Healthcare) column and the adsorbed proteins were eluted with eluting solution (buffer solution A containing 0.5 M imidazole). The obtained eluate was dialyzed with 20 mM Tris-HCl (pH 8.0) containing 50 mM NaCl as the external solution.

### Enzyme activity and inhibition by DMAPP, GPP, FPP and DPM

The catalytic activities of the MVKs were measured using a modified spectrophotometric assay that couples ADP formation to reverse pyruvate kinase and lactate dehydrogenase reactions [21]. The initial rate of disappearance of NADH served as a measure of the phosphorylation of mevalonate by MVK. The NADH millimolar extinction coefficient of 6.22 mM^−1^ cm^−1^ was used in this study.

Each 100 -µl reaction mixture contained 50 mM Tris (pH 7.6), 50 mM NaCl, 0.4 mM phosphoenol pyruvate, 0.05 mM DTT, 0.33 mM NADH, 10 mM MgCl_2_, 2 units of LDH and 2 units of PK.

The Michaelis constants with respect to mevalonate *K*_m__-Mev_ for MVK from *M. concilii*, *M. paludicola*, *M. mazei*, *N. maritimus* and *S. cerevisiae* were determined at a saturating concentration of ATP (5 mM) and variable concentrations of potassium mevalonate. The Michaelis constants to ATP *K*_m__-ATP_ were determined at a saturating concentration of potassium mevalonate (5 mM) and variable concentrations of ATP. *K*_m_ and *k*_cat_ values were determined by fitting the Michaelis–Menten equation using SigmaPlot 13.0/Enzyme Kinetics Module 1.3 (Systat Software, Inc.). The error values represent the standard deviation from three replicates. Changes in absorbance associated with the amount of NADH oxidized to NAD^+^ were monitored continuously at 340 nm and plotted against time to determine the rate of the MVK-coupled reactions.

Protein inhibition studies were performed by adding terpenyl diphosphates (DMAPP, GPP, FPP, or diphosphomevalonate) at various concentrations to the reaction mixture.

## Results

### Exploring the candidate *mvk* genes

The first feedback-resistant MVK was found in the methanogenic archaeon *Methanosarcina mazei* [23]. Methanogenic archaea are divided into seven distant taxonomic orders [28]. The majority of methanogenic archaea use CO_2_+H_2_ as substrates but cannot utilize acetate. Only two genera belonging to *Methanosarcinales*, *Methanosarcina* and *Methanosaeta* are able to utilize acetate for growth and methane production [29]; they are responsible for a major part of methane production in the biosphere.

Primak *et al*. demonstrated a clear evolutionary separation of MVKs from *Archaea*, *Bacteria* and *Eukarya* [23]. They also proposed that alternative regulatory mechanisms are inherent for different MVK phylogenetic branches.

We analysed MVKs from representatives of the class *Methanomicrobia*. Homologues of the *M. mazei* MVK were chosen from the database of non-redundant protein sequences of *Methanomicrobia* (taxid: 224756) using the psi-blast program [30]. clustalw [31] was used for multiple alignment, and a phylogenetic tree of the chosen proteins was constructed using the neighbour-joining (NJ) method [32]. Unexpectedly, the proteins from the genus *Methanosaeta* formed a distinct evolutionary branch that was proven to be more distant from the *Methanosarcina* branch than *Methanocellales* and *Methanomicrobiales* (Figs S1 and S2, available with the online Supplementary Material). Putative MVKs from *M. paludicola* SANAE and *M. concilii* GP6 were chosen for further analysis; both *M. paludicola* SANAE and *M. concilii* GP6 are mesophilic micro-organisms of biosafety level 1. Complete annotated genome sequences are available for both strains. *M. concilii* is a well-studied methanogenic bacterium commonly observed in digested sludge [33–35]. *M. paludicola* was the first cultivated representative of *Methanocellales* isolated from rice field soil [36, 37]. *M. paludicola* is unrelated to *M. mazei*, but it possesses similar MVK. Conversely, *M. concilii* belongs to the same family as *M. mazei*, but the MVK from *M. concilii* is more evolutionarily distant. We chose MVK from *N. maritimus*, which is classified in the phylum *Thaumarchaeota*, as an outgroup of MVK from *Methanomicrobia* [38].

### Cloning of the *mvk* genes

Nucleotide sequences of *mvk* genes from *M. concilii* GP6 (GenBank accession number: NC_015416.1, locus_tag=MCON_2559)*, M. paludicola* SANAE (GenBank accession number: AP011532.1, locus_tag=MCP_1639)*, M. mazei* Go1 (GenBank accession number: NC_003901.1, locus_tag=MM_RS09140) and *N. maritimus* (GenBank accession number: CP_000866.1, locus_tag=NMAR_0315), and of the ERG12 gene from *Saccharomyces cerevisiae* (GenBank accession number: X55875.1), were modified for efficient cloning and expression in *E. coli*. For the *mvk^mcl^*, *mvk^mpd^* and *mvk^mma^* genes, codon optimization (the substitution of codons that are rare in *E. coli* with synonymous codons that are more frequently used) was performed using OptimumGene provided by GenScript. The resultant nucleotide sequences are shown in Fig. S3 [39]. The DNA fragments were cloned into pET-21a(+) (C-terminal His-tag) and pET-28b(+) (N-terminal His-tag) expression vectors (Novagene), as described in the Methods section.

### Purification of MVKs

All constructed plasmids were transformed into the *E. coli* BL21(DE3) strain. To improve the solubility of MVKs, cultivation of the transformants was carried out at a low temperature (20 °C). The expression levels provided by the pET-21a(+)- and pET-28b(+)-derived plasmids were compared on the basis of the MVK activity assay (see the Methods section). In all cases, the pET-28b(+)-derived plasmids yielded higher or equal activity levels compared to derivatives of pET-21a(+). Thus, the pET-28b(+)-derived plasmids were chosen for further study. These plasmids provided the accumulation of the target recombinant proteins, which contained 20 additional amino acid residues, including a cluster of 6 His residues fused to the N-terminus of MVK, to simplify the purification process.

The recombinant MVKs from *M. concilii*, *M. paludicola*, *M. mazei*, *N. maritimus* and *S. cerevisiae* were purified using immobilized metal ion affinity chromatography (see the Methods section) up to >95 % apparent homogeneity, as determined based on SDS-PAGE and Coomassie staining.

MVKs belong to the GHMP kinase family [40]. Monomeric or homodimeric assembly was observed for the previously described members of this protein family. The apparent masses of MVKs from *M. concilii* and *M. paludicola* were determined by gel filtration to be 158 and 72 kDa, respectively. The corresponding molecular masses calculated from the primary amino acid sequences were 35.6 and 33.9 kDa, respectively. These results suggest that MVK*^mpd^* forms a dimer in solution, similar to other homodimeric GHMP kinases, while MVK*^mcl^* has an unusual tetrameric structure (Table S1).

### Kinetic characterization of MVKs

For each enzyme, the rates of mevalonate phosphorylation were monitored in coupled reactions with pyruvate kinase and lactate dehydrogenase according to [21] (see the Methods section). The obtained [S]-v plots are indicated in Fig. S4. The apparent *K*_m_ values with respect to ATP (*K*_m__-ATP_) and mevalonate (*K*_m__-Mev_) were calculated for each His-tagged recombinant enzyme using the Michaelis–Menten equation.

Under the experimental conditions, significantly higher affinity to mevalonate and ATP was observed for the MVKs from *M. concilii* (K_m-Mev_=17.0±0.5 µM, K_m-ATP_=74±1 µM) and *M. paludicola* (K_m-Mev_=15.0±0.5 µM, K_m-ATP_=119±2 µM) relative to MVK***^mma^*** (K_m-Mev_=83±2 µM, K_m-ATP_=687±9 µM) or MVK***^sce^*** (K_m-Mev_=73±2 µM, K_m-ATP_=464±8 µM). MVK***^mpd^*** exhibited the lowest turnover (*k***_cat_**=7.30±0.04 s^−1^) among all the studied enzymes, while MVK***^mcl^*** had a *k***_cat_** that was comparable to that of MVK***^mma^*** and MVK***^sce^*** (14.0±0.1 s^−1^, 19.0±0.2 s^−1^ and 16.0±0.1 s^−1^, respectively). The highest catalytic efficiency (*k*_cat_/K_m-Mev_=0.824 µM s^−1^) was observed for MVK***^mcl^*** (Table 2).

**Table 2.** Kinetic characterization of MVKs

MVK	K_m-Mev_ (μM)	K_m-ATP_ (μM)	MW (Da)	Specific activity µmol/min*mg	*k*_cat_ (s^−1^)	*k*_cat_ /K_m-Mev_ (µM/s)
*mcl*	17.0±0.5	74±1	35 626	233±2	14.0±0.1	0.824
*mpd*	15.0±0.5	119±2	33 908	129±1	7.30±0.04	0.487
*mma*	83±2	687±9	33 575	348±3	19.0±0.2	0.229
*nmr*	461±7.5	1006±20	36 594	204±2	12±0.11	0.026
*sce*	73±2	464±8	50 622	188±3	16.0±0.1	0.219

### Conversion of mevalonate to phospho- and diphosphomevalonate in an *in vitro* system containing a purified MVK and an *S. cerevisiae* phosphomevalonate kinase

Mevalonic acid contains two hydroxyl groups (C3 and C5) and a C1 carboxyl group that can be phosphorylated. Mevalonate-5-phosphate is an intermediate of the classical mevalonate pathway. Products of the reactions catalyzed by two other characterized archaeal MVKs from *Methanosarcina mazei* and *Methanocaldococcus jannaschii* can be further converted into diphosphomevalonate by *S. cerevisiae* 5-phosphomevalonate kinase (PMK) [23]. On the other hand, two ATP:(R)-mevalonate 3-phosphotransferases were recently identified in the archaea *Thermoplasma acidophilum* [16] and *Picrophilus torridus* [41].

**Fig. 1.**
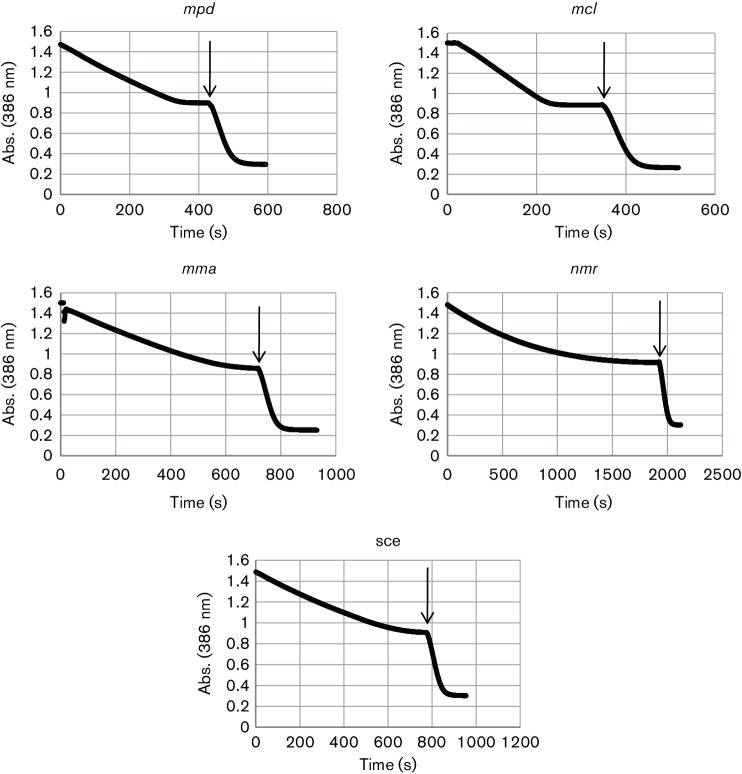
Conversion of phosphomevalonate produced by MVK*^mma^*, MVK*^sce^*, MVK*^mcl^*, MVK*^mpd^* and MVK*^nmr^* to diphosphomevalonate by *S. cerevisiae* PMK. The conversion of mevalonate to phospho- and, subsequently, diphosphomevalonate was monitored indirectly by the oxidation of NADH in a pyruvate kinase and lactate dehydrogenase-coupled assay. The kinetics of absorbance at 386 nm is plotted. Purified PMK was added at the points indicated by arrows.

To determine the group that is phosphorylated by MVK*^mcl^* and MVK*^mpd^*, we applied an *in vitro* assay previously developed by Andreassi *et al*. [21] and exploited in [23] to analyse the inhibition of MVKs by diphosphomevalonate. This approach couples the initial phosphorylation of mevalonate by MVK and the subsequent conversion of phosphomevalonate to diphosphomevalonate after PMK addition. In both cases, the rate of ADP formation was monitored as a decrease in absorbance at 386 nm in the spectrophotometric pyruvate kinase and lactate dehydrogenase-coupled assay (0.61 mM^−1^ cm^−1^ was designated as the millimolar absorption coefficient of NADH at 386 nm). The obtained curves confirmed the complete conversion of mevalonate to diphosphomevalonate via phosphomevalonate (Fig. 1). The obtained results strongly suggest that MVK*^mcl^* and MVK*^mpd^* should be considered as mevalonate-5-kinases. Nevertheless, to make sure that these enzymes cannot also produce mevalonate-3-phosphate, the direct identification of products formed in reactions with MVK*^mcl^* and MVK*^mpd^* is necessary.

In the reactions with MVK*^mma^*, MVK*^sce^*, MVK*^mcl^* and MVK*^mpd^*, an almost linear decrease in absorbance was observed until PMK addition. In contrast, a stepwise decrease in the reaction rate associated with product accumulation was observed for MVK*^nmr^*. This result suggests the product inhibition of MVK*^nmr^* by phosphomevalonate.

### Archaeal MVKs are not inhibited by downstream intermediates of isoprenoid biosynthesis (DPM, DMAPP, GPP and FPP)

The feedback inhibition of the *M. concilii*, *M. paludicola*, *M. mazei* and *N. maritimus* MVKs by the downstream intermediates of isoprenoid biosynthesis (diphosphomevalonate (DPM), DMAPP, geranyl pyrophosphate (GPP) and farnesyl pyrophosphate FPP) was evaluated. Parallel spectrophotometric activity assays that contained a high concentration (indicated in Table 3) of the potential inhibitor or lacked inhibitors were performed.

**Table 3.** Influence of the potential inhibitors on mevalonate kinase activity nd, not determined. Results of single experiments are represented. Relative catalytic activity (%) of the enzymes is indicated. Activity without potential inhibitors was taken as 100 %.

MVK	DPM	DMAPP	GPP			FPP			Reference
	1 mM	5 mM	5 µM	10 µM	100 µM	5 µM	10 µM	100 µM	
*sce*	96	8	nd	nd	9	nd	nd	10	This work
*mma*	85	91	nd	nd	96	nd	nd	98	This work
*mcl*	99	122	nd	nd	104	nd	nd	105	This work
*mpd*	105	97	nd	nd	101	nd	nd	101	This work
*nmr*	97	95	nd	nd	100	nd	nd	92	This work
*mja*	nd	nd	58	45	nd	46	35	nd	[11]

Table 3 indicates the relative catalytic activity (%) of each enzyme. The activity in the reaction mixture without inhibitors was taken as 100 %. According to previously published data, *S. cerevisiae* MVK is strongly inhibited by 5 mM DMAPP, 0.1 mM GPP and 0.1 mM FPP, but is not inhibited by 1 mM DPM. The *M. mazei* MVK was not inhibited by 0.1 mM GPP or 0.1 mM FPP. A slight decrease in activity was observed for this enzyme when 1 mM DPM or 5 mM DMAPP was added. Neither MVK*^mcl^* nor MVK*^mpd^* was inhibited by 1 mM DPM, 5 mM DMAPP, 0.1 mM GPP or 0.1 mM FPP. Conversely, the enzyme from *M. concilii* was even slightly activated by 5 mM DMAPP (its activity increased by 20 % compared to that of the control). Lower concentrations (33.6 or 168 µM) of DMAPP or IPP did not affect the activity of this enzyme.

## Discussion

Isoprenoids are produced by all organisms, and their precursors are known as essential compounds [42]. However, the redundant production of terpenoids or their precursors can be toxic for cells and cause various diseases [43, 44], which is why the strict control of isoprenoid biosynthesis is of vital importance. The regulation mode can be species-specific. Clarifying and comparing the biochemical features of archaeal MVKs is important for understanding the control mechanism.

The archaeal MVKs from *M. concilii* and *M. paludicola* characterized in this work were not inhibited by DPM, DMAPP, IPP, GPP or FPP, and exhibited very high affinity to mevalonate. The Michaelis constants for (R, S)-mevalonate determined for these enzymes are four- to fivefold lower than those for MVK*^mma^* (see [23] and this work) and MVK*^nmr^* (this work), or for the MVK from *M. jannaschii*, a hyperthermophilic, autotrophic and strictly hydrogenotrophic archaeon [11]. A similar K**_m_** for (R, S)-mevalonate K_m-Mev_=19.0 µM was previously observed for pig liver MVK; however, this enzyme is feedback-sensitive [45].

MVK*^mcl^* has proven to be an unusual enzyme. It is not inhibited by phosphomevalonate, DPM, GPP or FPP, and its activity is even increased by the addition of DMAPP. Unlike all previously characterized MVKs (Table S1), it has a tetrameric structure. Moreover, the conserved motif 4 of MVK*^mcl^* contains two positively charged amino acids – lysine and arginine – that are located between the residues involved in binding of the phosphate moiety of ATP (Fig. S5, triangles). This structural feature may cause the specific biochemical features of MVK*^mcl^*.

The biochemical properties of MVK*^mcl^* and MVK*^mpd^* make them promising candidates for industrial applications to promote isoprenoid compound production through the mevalonate pathway. In particular, our previous experiments confirmed the advantages of MVK*^mcl^* for isoprene production [39].

**Fig. 2.**
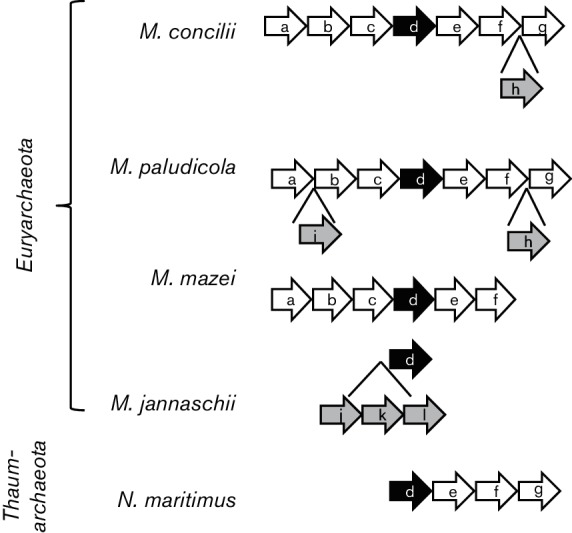
Conserved strings include genes encoding the feedback-resistant mevalonate kinases in *Archaea*. (a) RpoK, (b) 30S ribosomal protein S2, (c) hypothetical protein, (d) feedback-resistant mevalonate kinase, (D) feedback-sensitive mevalonate kinase, (e) isopentenyl kinase, (f) isopentenyl pyrophosphate isomerase, (g) bifunctional short chain isoprenyl diphosphate synthase, (h) beta-lactamase domain-containing protein, (i) enolase, (j) shikimate 5-dehydrogenase, (k) hypothetical protein and (l) hypothetical protein.

The tree topology of MVK differs from its representative phylogeny (Fig. S1), suggesting the existence of different biochemical features. Surprisingly, the three MVKs isolated from representatives of different families belonging to *Methanomicrobia* have proven to be feedback-resistant. Based on these results, we proposed that these organisms might use another mechanism (or other mechanisms) that is (are) distinct from the feedback system to regulate the mevalonate pathway. To elucidate whether this regulation could be realized at the level of transcription, we analysed the surroundings of the *mvk* orthologues found in the sequenced genomes using Search Tool for the Retrieval of Interacting Genes/Proteins (STRING) v.10 software [46]. All representatives of *Methanomicrobia* harbour long operons, including three genes of the lower mevalonate pathway coding for MVK, IP kinase and IPP isomerase (Fig. 2). It is likely that the expression of genes involved in the lower mevalonate pathway is co-regulated in these organisms at the transcriptional level. In many other *Euryarchaeota*, the *mvk* gene is linked to the IP kinase gene, as shown in Fig. 2. Conversely, an ORF encoding the previously characterized archaeal feedback-sensitive MVK from *M. jannaschii* is arranged in the genome as an individual gene. Further investigation is necessary to reveal the regulatory mechanisms that control isoprenoid biosynthesis in organisms harbouring feedback-resistant MVKs, and in addition, if a strong correlation between MVK inhibition and the genome arrangement of the corresponding ORF does exist, analysis of the genetic surroundings of *mvk* genes could help us explore novel feedback-resistant MVKs.

We hope that the obtained recombinant feedback-resistant MVK proteins will facilitate future studies on structure–function relationships. Previously, an X-ray structure of rat MVK in complex with farnesyl thiophosphate or ATP has been resolved; the amino acid residues that interact with the phosphate moieties of ATP or farnesyl thiophosphate have been determined [47]. The structure-based alignment of MVKs from organisms belonging to different domains of life (Fig. S5) revealed the strict conservation of these residues. On the other hand, among the residues of rat MVK involved in the binding of the isoprenoid moiety of farnesyl thiophosphate, only P139 is highly conserved; four residues are located in conserved regions, and five residues are located in a variable spacer between the conserved motifs 1 and 2. In prokaryotic MVKs, this spacer is considerably shorter than in eukaryotic enzymes. Thus, it is likely that the structure of this region determines the spectrum of inhibitors that can be bound by a concrete MVK. Further study of archaeal MVKs and their comparison with bacterial and eukaryotic enzymes can provide new insights into the mechanism of interaction between potential inhibitors and prokaryotic and eukaryotic MVKs. Such an investigation would provide interesting ideas for the relief of MVK inhibition in patients with autoinflammatory disorders caused by a mevalonate kinase (MK) deficiency [48] and for the design of new MVK inhibitors, which are being considered as potential drugs for the treatment of cardiovascular diseases and cancer [49] and as antibacterial agents [50].

### Conclusion

In conclusion, cloning, expression, purification, kinetic parameter determination and inhibition analysis of the MVKs from the mesophilic archaea *M. concilii* and *M. paludicola*, as well as from the marine thaumarchaeote *N. maritimus*, were performed. Consistent with the previously reported MVK from *M. mazei*, the new enzymes were not inhibited by downstream metabolites (DPM, DMAPP, IPP, GPP and FPP). At the same time, the MVKs from *M. concilii* and *M. paludicola* exhibited significantly higher affinity to mevalonate and higher catalytic efficiency than the previously characterized enzyme.

## Supplementary Data

1157 Supplementary File 1
