# Supplementary material for: Characterization of feedback-resistant mevalonate kinases from the methanogenic archaeons Methanosaeta concilii and Methanocella paludicola
Source: Microbiology (Reading). 2017 Sep 5;163(9):1283–91. doi: 10.1099/mic.0.000510 (PMC5817203; doi:10.1099/mic.0.000510)

FIGURE S1. Phylogenetic analysis of the 16S rRNA sequences and mevalonate kinase proteins of representative archaea in the class *Methanomicrobia* examined in this study

(a). Phylogenetic tree of the 16S rRNA sequences of representative archaea in the class *Methanomicrobia* examined in this study.

M. mazei, *M. concilii*, *M. paludicola*, *Methanocorpusculum bavaricum*, *M. jannaschii*, and *N. maritimus*, which are representative species of *Methanosarcinaceae*, *Methanosaetaceae*, *Methanocellaceae*, *Methanocorpusculaceae*, *Methanocaldococcaceae* and *Nitrosopumilaceae* families, respectively. The class *Methanomicrobia* contains the orders *Methanosarcinales*, *Methanocellales* and *Methanomicrobiales*. *Methanosarcinaceae* and *Methanosaetaceae* belong to the order *Methanosarcinales*, *Methanocellaceae* belongs to the order *Methanocellales*, and *Methanocorpusculaceae* belongs to the order *Methanomicrobiales*. *M. jannaschii* and *N. maritimus* were used as outgroups. *M. bavaricum* diverged from a common ancestor of class *Methanomicrobia*; *M. paludicola* diverged from a common ancestor of orders *Methanosarcinales* and *Methanocellales*. The numbers indicate bootstrap values for 1000 replications. 16S rRNA sequences were obtained from DDBJ/EMBL/GenBank database or RefSeq (ID shown): AUMX01000033, from 12 to 1465: *M. bavaricum* DSM 4179; CP002565, from 291377 to 292844: *M. concilii* GP6; AE008384, from 235306 to 236779: NC_000909 from 157985 to 159459: *M. jannaschii* DSM 2661; *M. mazei* Go1; AP011532, from 106585 to 108054: *M. paludicola* SANAE; CP000866, from 896241 to 897709: *N. maritimus* SCM1.

(b). Phylogenetic tree of the MVK protein sequences of representative archaea in the class *Methanomicrobia* examined in this study.

M. jannaschii and *N. maritimus* were used as outgroups. *M. concilii* diverged from a common ancestor of *M. bavaricum*, *M. paludicola* and *M. mazei*. The numbers indicate bootstrap values for 1000 replications. MVK protein sequences were obtained from DAD/TrEMBL/GenPept database (ID shown): WP_042697404.1: *M. bavaricum* DSM 4179; WP_013720012.1: *M. concilii* GP6; AAB99088.1: *M. jannaschii* DSM 2661; AAM31458.1: *M mazei* Go1; BAI61711.1: *M. paludicola* SANAE; ABX12211.1: *N. maritimus* SCM1.

(a)

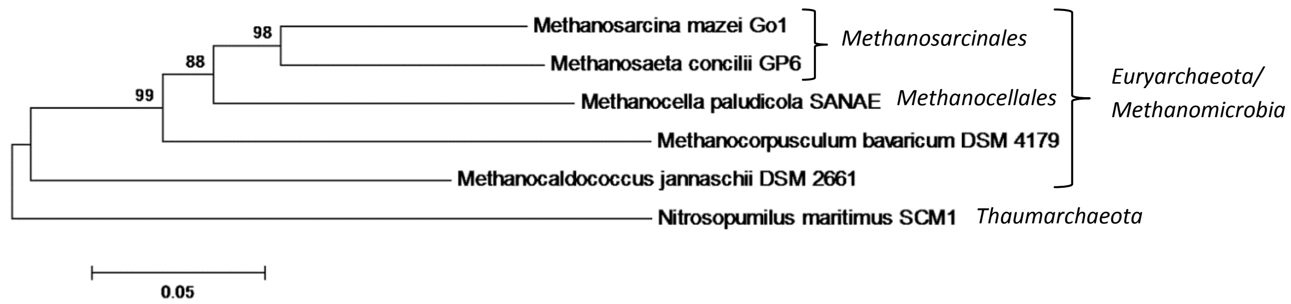
98
88
99
Methanosarcina mazei Go1
Methanosaeta concilii GP6
Methanosarcinales
Methanocella paludicola SANAE
Methanocellales
Methanocorpusculum bavaricum DSM 4179
Methanocaldococcus jannaschii DSM 2661
Euryarchaeota/
Methanomicrobia
Nitrosopumilus maritimus SCM1
Thaumarchaeota
0.05

(b)

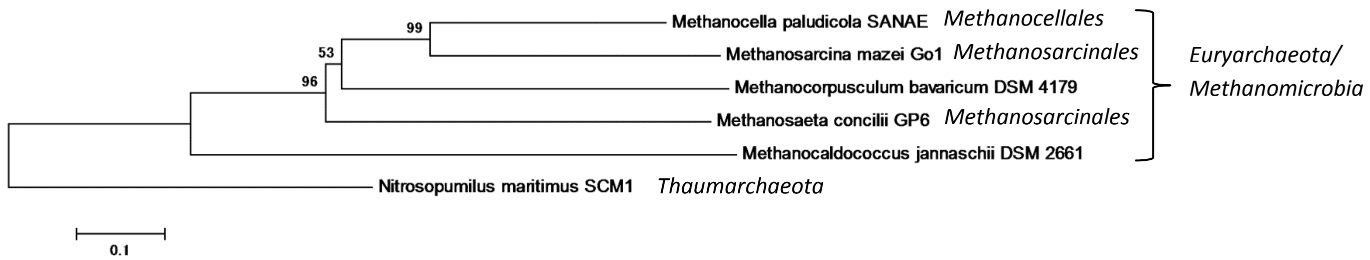
99
53
96
Methanocella paludicola SANAE
Methanocellales
Methanosarcina mazei Go1
Methanosarcinales
Methanocorpusculum bavaricum DSM 4179
Methanosaeta concilii GP6
Methanosarcinales
Methanocaldococcus jannaschii DSM 2661
Euryarchaeota/
Methanomicrobia
Nitrosopumilus maritimus SCM1
Thaumarchaeota
0.1

FIGURE S2. Phylogenetic tree of mevalonate kinase proteins.

N. maritimus was used as out group. The numbers indicate bootstrap values for 1000 replications. Amino acid sequences for the following proteins were obtained from the DAD/TrEMBL/GenPept database (ID shown): WP_012037131.1: *Methanocella arvoryzae*; WP_014406849.1: *Methanocella conradii*; BAI61711.1: *Methanocella paludicola* SANAE; WP_011500381.1: *Methanococcoides burtonii*; WP_048193216.1: *Methanococcoides methylutens*; WP_042697404.1: *Methanocorpusculum bavaricum*; WP_011833054.1: *Methanocorpusculum labreanum*; WP_013037987.1: *Methanohalophilus mahii*; WP_048150253.1: *Methanolacinia paynteri*; WP_013328711.1: *Methanolacinia petrolearia*; WP_015052815.1: *Methanolobus psychrophilus*; WP_023846035.1: *Methanolobus tindarius*; WP_042705532.1: *Methanomicrobium mobile*; WP_004076515.1: *Methanoplanus limicola*; WP_013720012.1: *Methanosaeta concilii*; WP_014586538.1: *Methanosaeta harundinacea*; ABK14267.1: *Methanosaeta thermophila* PT; AAM04046.1: *Methanosarcina acetivorans* C2A; WP_048154424.1: *Methanosarcina barkeri*; WP_054297850.1: *Methanosarcina flavescens*; WP_048124443.1: *Methanosarcina lacustris*;WP_048036269.1: *Methanosarcina mazei*; WP_048179583.1: *Methanosarcina siciliae*; WP_048051657.1: *Methanosarcina soligelidi*; WP_048153542.1: *Methanosarcina* sp. Kolksee; WP_048167857.1: *Methanosarcina thermophile*; WP_048117800.1: *Methanosarcina vacuolata*; WP_042685946.1: *Methermicoccus shengliensis*; ABX12211.1: *Nitrosopumilus maritimus*

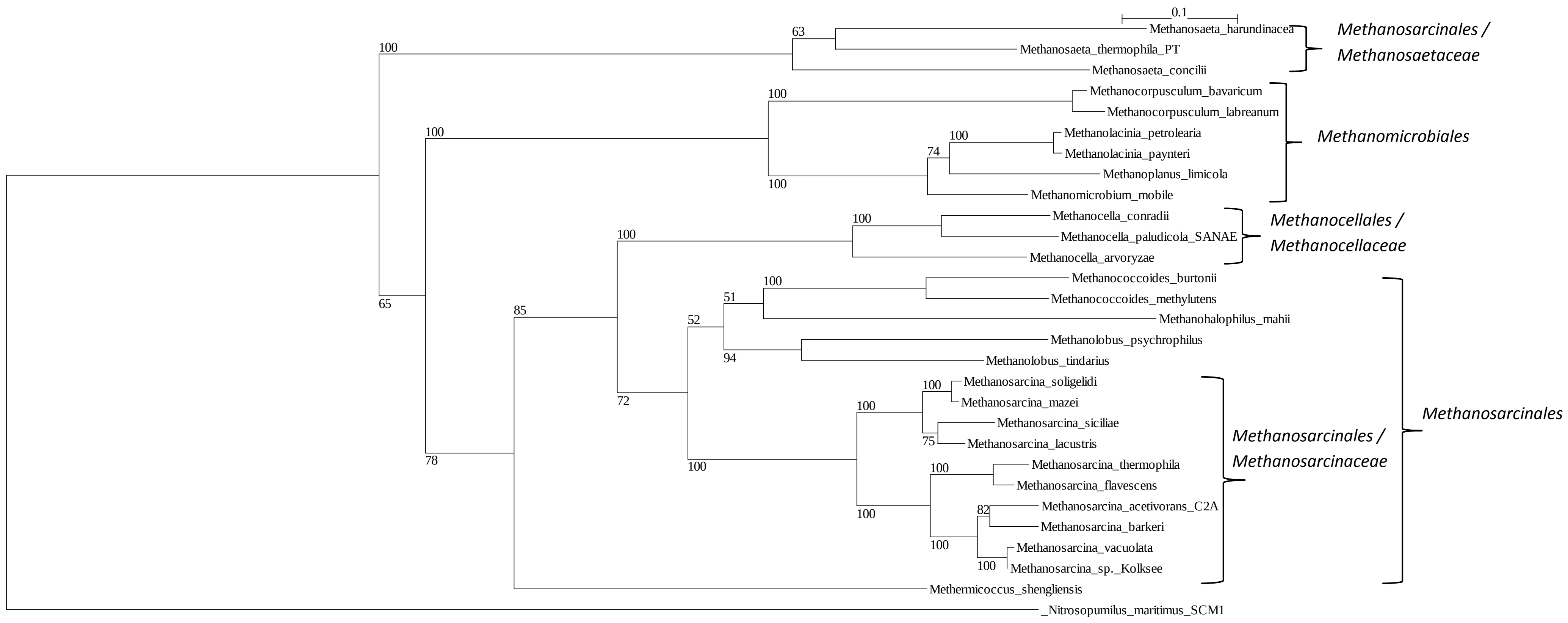
0.1
Methanosaeta_harundinacea
Methanosaeta_thermophila_PT
Methanosaeta_concilii
Methanocorpusculum_bavaricum
Methanocorpusculum_labreanum
Methanolacinia_petrolearia
Methanolacinia_paynteri
Methanoplanus_limicola
Methanomicrobium_mobile
Methanocella_conradii
Methanocella_paludicola_SANAE
Methanocella_arvoryzae
Methanococcoides_burtonii
Methanococcoides_methylutens
Methanohalophilus_mahii
Methanolobus_psychrophilus
Methanolobus_tindarius
Methanosarcina_soligelidi
Methanosarcina_mazei
Methanosarcina_siciliae
Methanosarcina_lacustris
Methanosarcina_thermophila
Methanosarcina_flavescens
Methanosarcina_acetivorans_C2A
Methanosarcina_barkeri
Methanosarcina_vacuolata
Methanosarcina_sp._Kolksee
Methermicoccus_shengliensis
_Nitrosopumilus_maritimus_SCM1
Methanosarcinales / Methanosaetaceae
Methanomicrobiales
Methanocellales / Methanocellaceae
Methanosarcinales / Methanosarcinaceae
Methanosarcinales

FIGURE S3. The chemically synthesized gene with optimized codons encoding MVK$^{\boldsymbol{mpd}}$.

1 ATGACGATGT GCTCAGCTCC GGGTAAAGTT TTTCTGTTTG GTGAACACGC
AGTGGTCTAT GGTAAACGCG CAATCGCCTG TGCTATCGAT CTGCGTACCA
101 CGGTCGAAGT GTCTCGTAAA AGTCGCGGTG TGCATATTCA CAGCGCGTTT
AAAGATGAAC CGGACAAAAA CCTGTATATC AAAACCGCAG TTAAACGTAT
201 GCAGAAATGT GCTGATATTC GCAACGTCAA TATCGCCGTG AGCTCTCGCA
TTCCGGTTGC CAGTGGCCTG GGTAGTTCCG CGGCCGTTAC CGTCGCGACG
301 ATTGGTGCCC TGAATGAAGA ATTTTCCGCC GGCCTGAGCA AAAAAGATAT
CGCGTATATG GCCTACCAGA CCGAACTGGA AGTTCAAGGT GCAGCTTCTC
401 CGACCGATAC GTTCGTCAGT ACCATGGGCG GTACGGTGGT TGTCCCGGAC
ATGCGTACCC TGCCGCCGAT TACGTGCGGC ATCGTGGTTG GCCATACCGG
501 TATTTCAAAA TCGACGAGCC GCATGGTTAG CCGTGTTCGC ACCCTGAAAG
AAAAATATCC GGATGTCATT GACGGCATCA TGGATTCGAT TGGTGACATC
601 AGCGCACGTG GCGAAGATCT GATTAAACAG AACGACTACC GCTCCATCGG
CGAACTGATG AACGTGAATC AAGGTCTGCT GGATGCCCTG GGCATTACCA
701 TCCCGGAACT GTCTCTGCAG ATCTATGCGG CCCGTCAACA CGGCGCATAC
GGTGCTAAAA TTACGGGTGC AGGCGGTGGC GGTTGCATGG TGGCTATCTG
801 TGATGACAAA AACTGCAAAG AAATTGCAAC CGCTATCGGC CGCTCGTATG
GCGACTCGTT CATCAGCAAA CCGACGGCGG AAGGTATTTT CATTCAGTGA

FIGURE S4. The Michaelis-Menten plots for the calculation of Km

Mcl

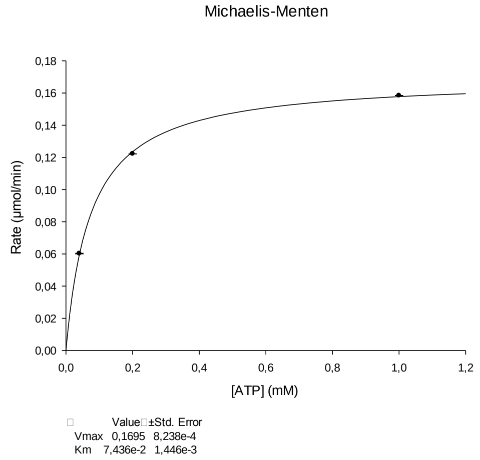

Mma

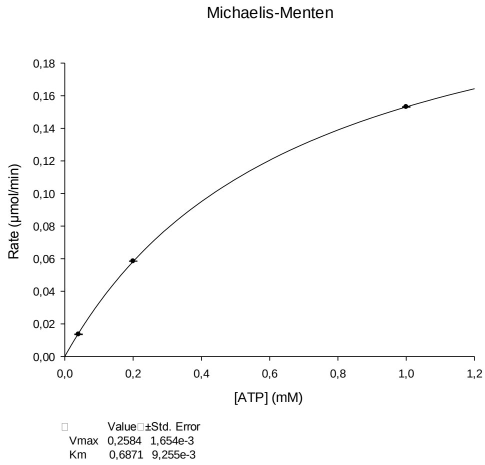

Nmr

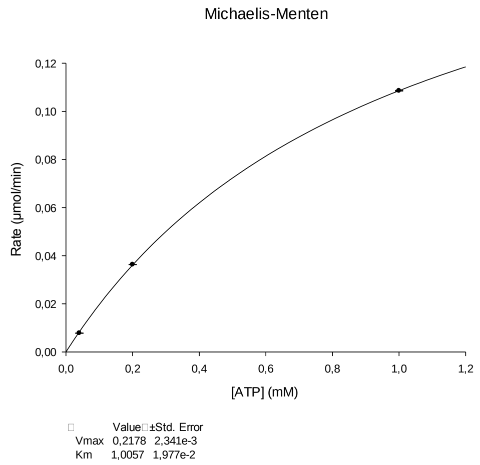

Sce

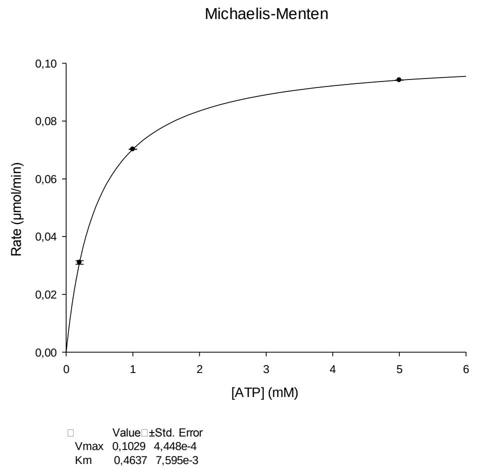

Mpd

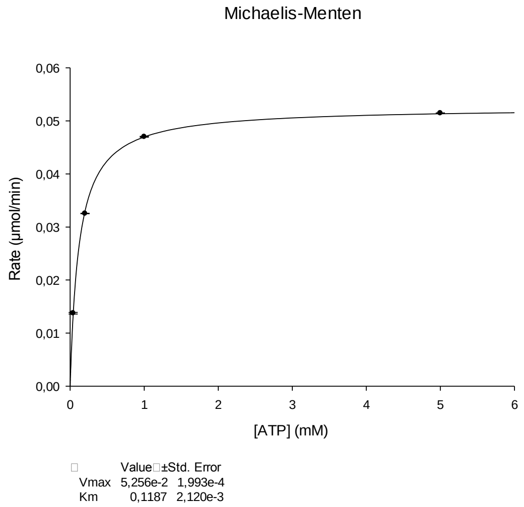

Mcl

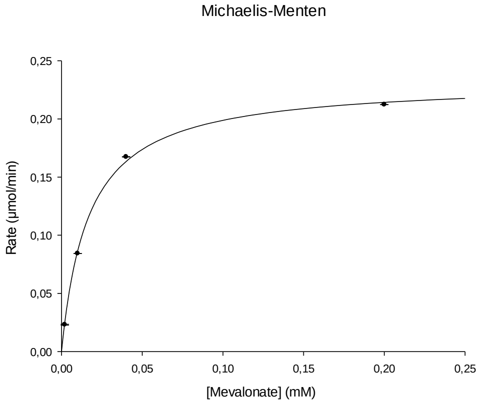

	Value	±Std. Error
Vmax	0,2323	2,060e-3
Km	1,683e-2	5,451e-4

Mma

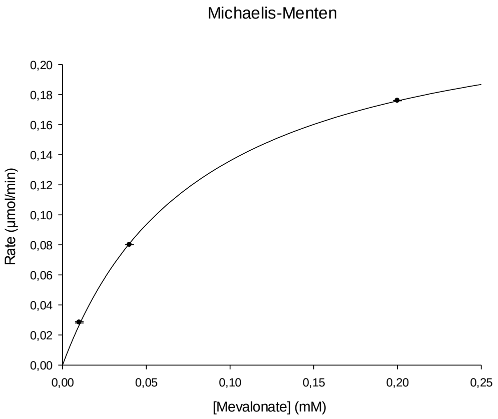

	Value	±Std. Error
Vmax	0,2487	2,324e-3
Km	8,294e-2	1,922e-3

Nmr

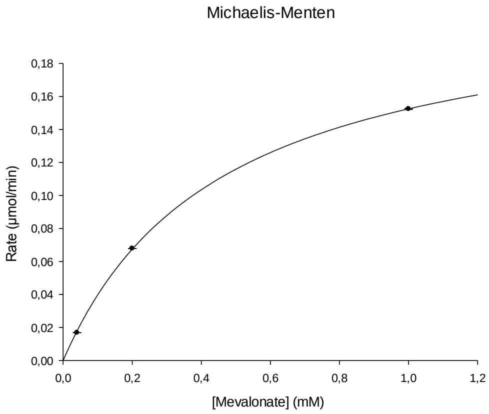

	Value	±Std. Error
Vmax	0,2227	1,500e-3
Km	0,4606	7,515e-3

Sce

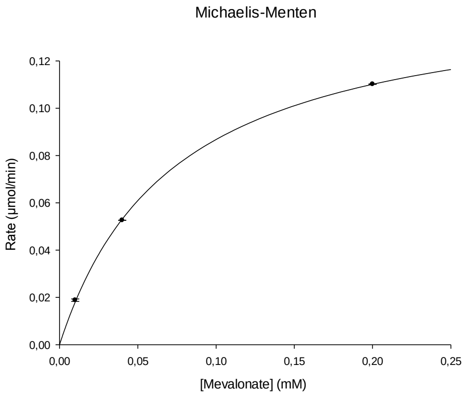

	Value	±Std. Error
Vmax	0,1506	1,262e-3
Km	7,355e-2	1,584e-3

Mpd

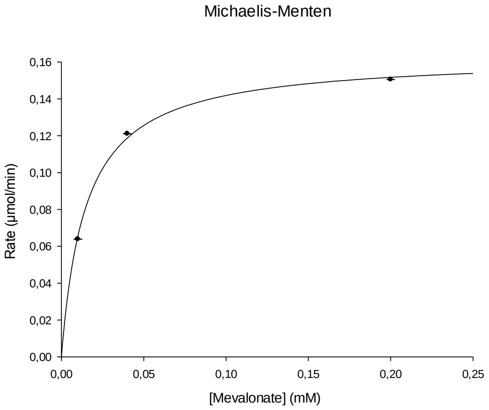

	Value	±Std. Error
Vmax	0,1630	1,430e-3
Km	1,489e-2	5,131e-4

TABLE S1. Subunit structure of mevalonate kinases (data extracted from the BRENDA data base)

Organism	Molecular weight, kDa		Citation
	Intact protein*	Subunit weight**	
H. sapiens	78	42.5	[1]
R. norvegicus	86	42.0	[2]
Sus scrofa	98.0	52.0	[3]
Gallus gallus	96.6-100.0	41.8	[4]
H. brasiliensis	98.0-102.0	40.9	[4]
Phaseolus vulgaris	97.0-104.0	nd	[5]
Cucumis melo	99.8-103.5	nd	[5]
Catharanthus roseus	101.9	41.5	[6]
S. cerevisiae	96.6-103.5	48.5	[4]
E. faecalis	32.2 / 71.1 (10%)	32.2	[7]
S. aureus	34.5	33.0	[8]
M. mazei	64.6	35.5	[9]
M. janaschii	68.0	37.0	[10]

* - Gel filtration or sucrose gradient data; **** -** deduced from nucleotide sequence or SDS PAGE data

1. **Potter D, Miziorko HM.** Identification of catalytic residues in human mevalonate kinase. *J Biol Chem* 1997;272(41):25449-25454.
2. **Tanaka RD, Schafer BL, Lee LY, Freudenberger JS, Mosley ST**. Purification and regulation of mevalonate kinase from rat liver. *J Biol Chem* 1990;265(4):2391-8.
3. **Porter JW**. Mevalonate kinase. *Methods Enzymol* 1985;110:71-78.
4. **Gray JC, Kekwick RG**. An assessment of some molecular parameters of mevalonate kinase from plant and animal sources. *Arch Biochem Biophys*. 1973;159(1):458-462.
5. **Gray JC, Kekwick RG.** Mevalonate kinase in green leaves and etiolated cotyledons of the french bean *Phaseolus vulgaris. Biochem J*. 1973;133(2):335-347.
6. **Schulte AE, van der Heijden R, Verpoorte R**. Purification and characterization of mevalonate kinase from suspension-cultured cells of *Catharanthus roseus* (L.) G. Don. Arch *Biochem Biophys* 2000;378(2):287-298.
7. **Hedl M, Rodwell VW.** *Enterococcus faecalis* mevalonate kinase. *Protein Sci* 2004;13(3):687-693.
8. **Voynova NE, Rios SE, Miziorko HM**. Staphylococcus aureus mevalonate kinase: isolation and characterization of an enzyme of the isoprenoid biosynthetic pathway. *J Bacteriol* 2004;186(1):61-67.
9. **Zhuang N, Seo KH, Chen C, Zhou J, Kim SW *et al.*** Crystallization and preliminary X-ray diffraction analysis of mevalonate kinase from *Methanosarcina mazei*. *Acta Crystallogr Sect F Struct Biol Cryst Commun* 2012;68(Pt 12):1560-1563.
10. **Huang KX, Scott AI, Bennett GN.** Overexpression, purification, and characterization of the thermostable mevalonate kinase from *Methanococcus jannaschii*. *Protein Expr Purif* 1999;17(1):33-40.

FIGURE S5. Structure-based sequence alignment of MVK proteins

The secondary structure of *Rattus norvegicus* MVK, which is placed above the alignment, was downloaded from the PDB database. Conserved residues in all four proteins are highlighted in black. Residues involved in the binding of the phosphate moieties of FSP (or ATP) are indicated by circles, and those involved in the isoprenoid moiety binding are denoted by stars. Structure-based amino acid sequence alignment was calculated and drawn with the CLUSTALX program and the ESPRIPT 3.0 web tool. Motifs 1, 2 and 3 – conserved GHMP motifs; Motif 4 – a less-conserved motif that can be defined on the basis of this alignment. Positively charged lysine and arginines in Motif 4 of MVK^{mcl} are indicated by triangles. Hsa – *Homo sapiens*, Rno – *Rattus norvegicus*, Hbr – *Hevea brasiliensis*, Efq – *Enterococcus faecalis*, Spn – *Streptococcus pneumonia*, CL190 – *Streptomyces* sp. CL190.

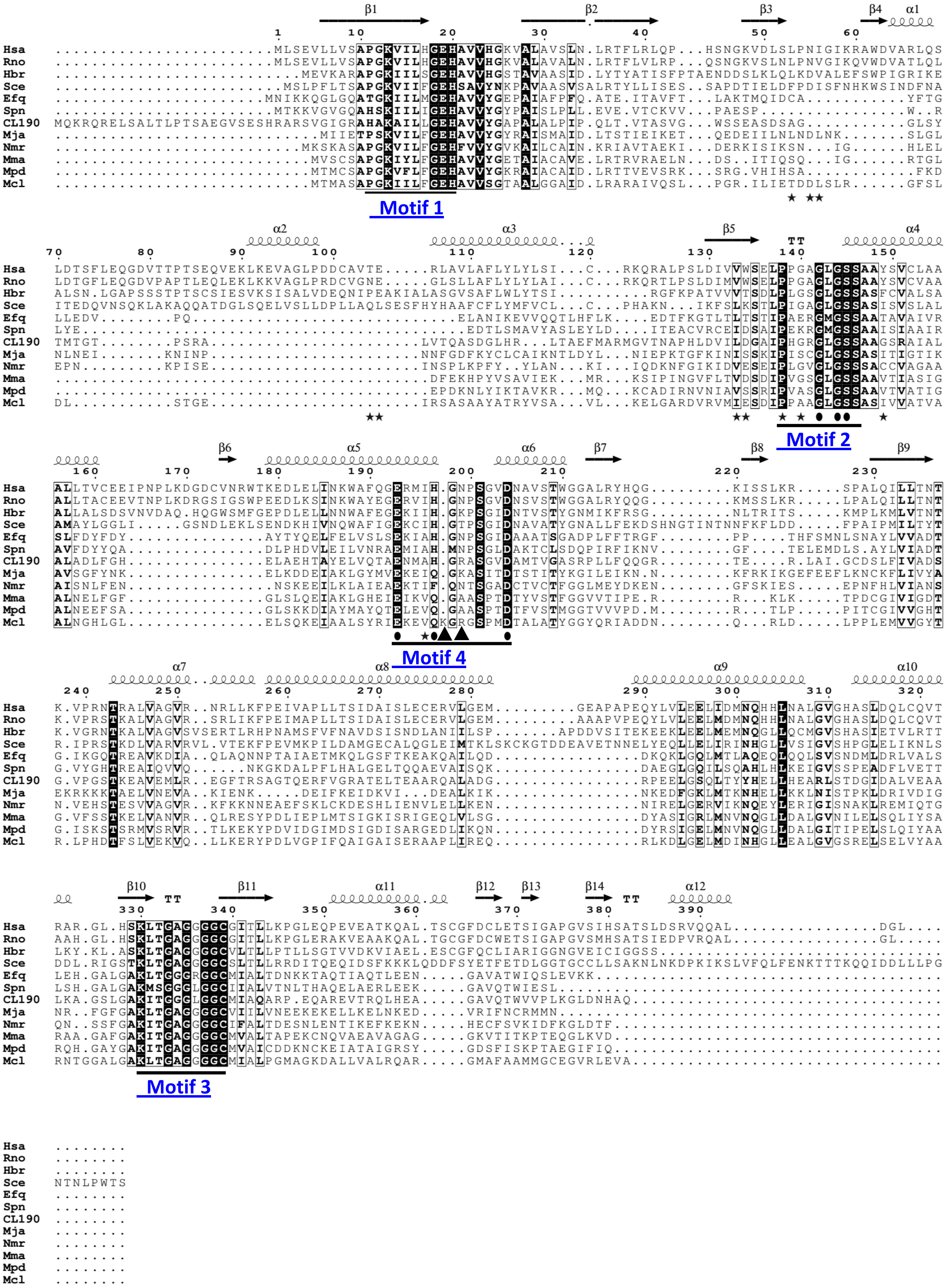

Supplement: Supplementary File 1 [file mic-163-1283-s001.pdf]
